# Compression Density as an Alternative to Identify an Optimal Moisture Content for High Shear Wet Granulation as an Initial Step for Spheronisation

**DOI:** 10.3390/pharmaceutics14112303

**Published:** 2022-10-26

**Authors:** Selina Ramm, Ruwen Fulek, Veronika Anna Eberle, Christian Kiera, Ulrich Odefey, Miriam Pein-Hackelbusch

**Affiliations:** 1 Department of Life Science Technologies, OWL University of Applied Sciences and Arts, Campusallee 12, 32657 Lemgo, Germany; 2 PHARBIL Pharma GmbH, Reichenbergerstr. 43, 33605 Bielefeld, Germany

**Keywords:** wet granulation, liquid requirement, granulation endpoint, compression density

## Abstract

Pellet production is a multi-step manufacturing process comprising granulation, extrusion and spheronisation. The first step represents a critical control point, since the quality of the granule mass highly influences subsequent process steps and, consequently, the quality of final pellets. The most important parameter of wet granulation is the liquid requirement, which can often only be quantitatively evaluated after further process steps. To identify an alternative for optimal liquid requirements, experiments were conducted with a formulation based on lactose and microcrystalline cellulose. Granules were analyzed with a Powder Vertical Shear Rig. We identified the compression density (*ρ*_press_) as the said alternative, linking information from the powder material and the moisture content (*R*^2^ = 0.995). We used *ρ*_press_ to successfully predict liquid requirements for unknown formulation compositions. By means of this prediction, pellets with high quality, regarding shape and size distribution, were produced by carrying out a multi-step manufacturing process. Furthermore, the applicability of *ρ*_press_ as an alternative quality parameter to other placebo formulations and to formulations containing active pharmaceutical ingredients (APIs) was demonstrated.

## 1. Introduction

In the pharmaceutical industry granulation is used as a process for size enlargement of small particles. For the granulation of pharmaceutical solids, wet granulation is often applied. This process starts with mixing dry powder, followed by wetting and nucleation once the liquid is added. The nuclei form the basis for the following granule growth and consolidation. If too much liquid is added, granules become a wet mass or slurry due to oversaturation [1,2]. It can thus be concluded that the moisture content is a key factor for the quality of granules [3]. The amount of liquid determines the degree of saturation in the granule structure. The saturation degree is defined by the percentage of intragranular voids filled with liquid and can be described by three states of granules. With increasing liquid loading, the structure changes from pendular, over funicular to capillary state as the saturation reaches 100% [2]. Beyond full saturation, powder particles become suspended in a continuous liquid phase [4].

A critical unit operation is the determination of liquid requirement as there is no intrinsic endpoint. In fact, the optimal endpoint depends on the desired properties of the granules [5], on process conditions and the applied equipment [1], and on the physical properties of the dry raw materials, such as solubility, surface area, particle shape and size distribution. These properties influence the packing pattern of the powder mixture and, thus, the degree of densification and, consequently, the saturation level of the mixture associated with the liquid requirement [2,6].

Therefore, many different approaches exist for determining the required moisture content for granules. An objective method is estimating the required liquid amount by applying a mathematical calculation. For this purpose, Leuenberger et al. [7] developed the following equation.

(1) $$\begin{array}{cc} \omega_{dry} & =\frac{\rho_{l}\;\cdot \;\varepsilon \;\cdot \;\bar{\gamma }}{\rho_{s}\;\cdot \;\left(1-\varepsilon \right)}+\delta \end{array}$$

In Equation (1), $\omega_{dry}$ is the required water content on a dry basis, ε is the porosity of the tapped powder, $\rho_{l}$ is the density of the liquid, $\rho_{s}$ is the true density of the solid. $\bar{\gamma }$ corresponds to a mean complete formation of liquid bridges in the pile and δ is the equilibrium moisture content of the product at 100% air humidity. Limitations for the use of this equation are, on the one hand, porosity ε to be estimated on the basis of the tapped density. On the other hand, for $\bar{\gamma }$, the packing of the particles to be either cubic or rhombohedral has to be estimated, as well as the degree of filling pore spaces. Furthermore, this calculation only provides the optimal moisture content related to the saturation level, but it does not consider the mechanical properties of the granules.

These properties are even more important if granules are further processed [8], for example, during tabletting or extrusion. In a multi-step manufacturing process, such as pellet production, granulation is followed by extrusion and spheronization. The liquid requirement of the initial granule mass thus directly correlates with the pellet quality [3]. The so-called hand pressure test is, for this reason, typically applied during formulation development. In this test, the granules are compressed by hand, and if the mass does not crumble, the amount of liquid is suitable for further processing [9,10,11,12]. The main disadvantage of this method is the subjectivity and the required experience needed to reproducibly perform this test. A scientifically sound upgrade of this method is the torque rheological characterization performed with a mixing torque rheometer [11,13,14,15]. The changes in torque thereby display the resistance of the granule mass relative to mixing. Plotting changes in torque against the binder ratio allows identifying different wetting phases during granulation. Moreover, the binder ratio yielding the maximum change in torque was identified as optimal [13,14,15]. This optimal value has also been shown to be relevant for extrusion–spheronization [11]. Indirectly, the liquid requirement can also be evaluated by physical properties of the granules, such as bulk and tapped density and the resulting Carr’s compressibility index [16,17,18]. The disadvantage is that the samples must first be dried before standard analytical methods; for example, sieving for particle sizing can be used.

In our work, we aimed to identify an alternative parameter that can predict the quality of the pellets already during granulation by means of the liquid requirement of the formulation. Based on a placebo formulation, a suitable alternative quality parameter, connected with the formulation’s composition and with the moisture content, should be identified. The applicability of this parameter should be verified by predicting liquid requirements for further formulation compositions. In addition, we wanted to carry out extrusion and spheronization based on predicted moisture contents and to discuss the quality of resulting pellets. Since formulations containing active pharmaceutical ingredients (APIs) are more commonly produced in the industry than placebo formulations, initial experiments should be conducted to assess whether the identified parameter could also be applied for API formulations.

## 2. Materials and Methods

The applied raw materials used in this study (lactose grades, mannitols, microcrystalline celluloses and APIs) are summarized in Table 1.

### 2.1. Granulation Equipment

Granulation was performed in a Thermomix^®^ TM6 (Vorwerk, Wuppertal, Germany) with a bowl size of 2.2 L. According to the manufacturer, the impeller’s speed can be adjusted from 40 rpm to 10,700 rpm [19]. In our study, we adjusted the impeller speed at 150 rpm. We replaced the original agitator by a custom-made mixing knife. The original Thermomix mixing knife was adapted by cutting off four mixing knives; then, stirring blades with dimensions of 4.5 cm × 1.5 cm × 0.2 cm were welded at an angle of 45°. This modification ensured proper material mixing and mimicked Diosna’s P 1–6 intensive mixer (DIOSNA Dierks & Söhne, Osnabrück, Germany). Water was added via a peristaltic pump (1B.1003-R/65, Petro Gas, Berlin, Germany). The inner diamater of the tube was 5 mm and equipped with a nozzle with an inner diameter of 1 mm. The amount of water added was controlled with a scale (PCE-BS 3000, PCE Instruments^™^, Meschede, Germany).

### 2.2. Granulation in Lab-Scale

A dry mass of 150 g was used for each batch. Dry materials were passed through a 1400 μm sieve and transferred to the mixing bowl. After dry mixing for 3 min in the Thermomix® TM6 (see Section 2.1) with an impeller speed of 150 rpm, water was added with 15 mL min^−1^, while keeping the impeller’s speed constant. The time for water addition was dependent on the amount of water. For example, with a dry mass of 150 g and a target water content on wet basis (*ω*_wet_) of 20%, the addition time was 2.5 min (37.5 g water). To ensure the preparation of homogeneous samples after all water was added, the mixing process stopped and material adhering to the mixing bowl was scraped off manually. Subsequently, larger agglomerates were crushed at a speed of 2000 rpm for 3 s. For each formulation, one batch was performed and samples were taken for the respective *ω*_wet_. The sample size was 38 g so that the compression density (*ρ*_press_) could be determined in triplicates (12 g each). The experimental procedure is summarized in Table 2. For details on the raw materials (abbreviated with A, B and API), we refer to Table 1.

### 2.3. Measurement of ρ_press_

A Texture Analyser (TA.XT*plus*, Stable Mirco Systems, Godalming, UK) with a 50 kg load cell and a Powder Vertical Shear Rig (A/PVS) was used to determine *ρ*_press_. For each analysis, 12 g of granulate was weighed in and transferred to the body of the rig and compressed with 196 N for 20 s. The final height of the compressed material was registered. For data recording and evaluation, software Exponent (Stable Mirco Systems, Godalming, UK) was used.

### 2.4. Proof of Concept: Pellet Production in Lab-Scale

For the multi-step manufacturing process of granulation, extrusion, spheronization and drying, a total dry mass of 500 g per batch was used. Granulation was carried out in the Bosch MUM6712 (Robert Bosch Hausgeräte GmbH, München, Germany) with a bowl size of 5.3 L, equipped with twin beating whisks. Bosch MUM6712 was qualified for accurate impeller speed (with a maximum deviation of ±3%) over the entire working range. After 5 min of dry mixing at agitator level 1 (impeller speed without load is 95 rpm; under load, the speed decreases), water was added by hand (for example, about 3 min for 200 g water), followed by another 5 min of mixing. Extrusion was performed using Schlüter Extruder PP85 (Maschinenfabrik H. Schlüter GmbH, Neustadt a. Rbge., Germany) with a MA9/085-003 die and 0.1 mm coller distance at 500 rpm. The screen size was 85 mm in diameter, and each bore had a diameter of 1.0 mm and a bore length of 2.0 mm. Schlüter RM300 (Maschinenfabrik H. Schlüter GmbH, Neustadt a. Rbge., Germany) was used as the spheronizer. Spheronization was performed at 434 rpm for 1 min, 665 rpm for 1 min, 786 rpm for 3 min and at 1028 rpm for 5 min. The diameter of the spheronizer disc was 300 mm. Drying was carried out overnight at 50 °C in the drying cabinet HORO 200 (HORO Dr. Hofmann GmbH, Ostfildern, Germany). The residual moisture content was determined with the Mettler Toledo Moisture Analyser HG83 (Mettler Toledo AG, Greifensee, Schweiz). In detail, sample sizes of 3 g pellets were measured at 105 °C. The measurement stopped as soon as the mean weight loss over 50 s was less than 1 mg. Determined residual moisture contents of the pellets are summarized in Table A2. Sieving for particle size distribution was performed by the manual removal of over- and under-sized pellets using 0.8/1.6 mm (1.6/4.0 mm) Kressner sieves. Sieve analysis of in sized fraction (0.8–1.6 mm) was performed with the JEL exzenter lab sieving machine (J. Engelsmann, Ludwigshafen, Germany). The sieve stack of 0.8/1.0/1.25/1.4/1.6 mm was loaded with 25 g for 5 min. For presentation, the results were combined into two fractions (0.8–1.6 mm and 1.6–4.0 mm).

### 2.5. Statistical Analysis

*ρ*_press_ measurements were performed in triplicates. Statistical analyses were performed by one-way ANOVA. A *p*-value smaller than 0.05 was considered statistically significant. Statistical analyses were performed using Minitab® 20.4. All experimental data are presented as mean ± standard deviation (*n* = 3).

## 3. Results and Discussion

### 3.1. Identification of a Moisture Related Quality Parameter for Granules

**Figure 1:**
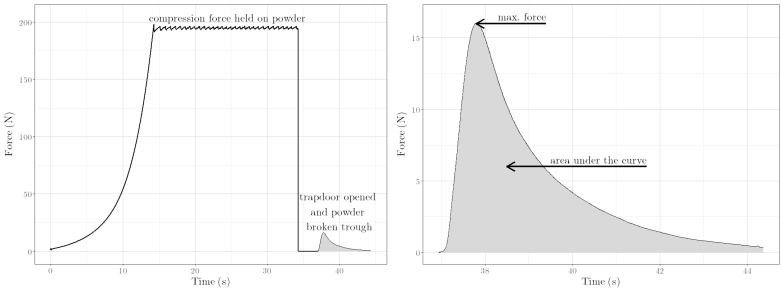
Measurement curve of the Powder Vertical Shear Test (formulation with 80:20 Lac 200:MCC 101 ratio and ω_wet_ 15%), performed with the Texture Analyser TA.XT*plus* with the Powder Vertical Shear Rig. (**Left**): Entire curve. (**Right**): Curve section, resulting from the shearing process. Highlighted in gray: Area under the curve.

The quality of the granule mass prior pelletisation is often determined by the hand pressure test and, thus, by the behavior of the granules under/after compression. We therefore utilized the at-line capable measuring method of the Texture Analyser with the Powder Vertical Shear Rig, which allowed the measurement of various parameters of the granulated material under compression and under shear. During the test, the powder is compressed under defined conditions (Section 2.3), yielding *ρ*_press_. In a subsequent step of the test, a trap door underneath the powder cake opened. A piston then pushes (powder shear speed 0.5 mm s^−1^, distance 5 mm) a plug of the powder cake through this opening. During the entire process, the drag force is recorded (Figure 1, left part). Here, the area under the curve, the maximum force and the vertical shear strength are monitored. The area under the curve is provided by the integral of the curve section resulting from the shearing process (Figure 1, area highlighted in gray) and the maximum force corresponds to the local maximum of this curve section (Figure 1 right part). The vertical shear strength is the ratio of the max. force and the lateral surface area of the briquette (Equation (2)). Detailed information about the described measurement has been published in [20].

(2) $$\begin{array}{cc} vertical\;shear\;strength & =\frac{max.\;force}{lateral\;\;surface\;\;area} \end{array}$$

**Figure 2:**
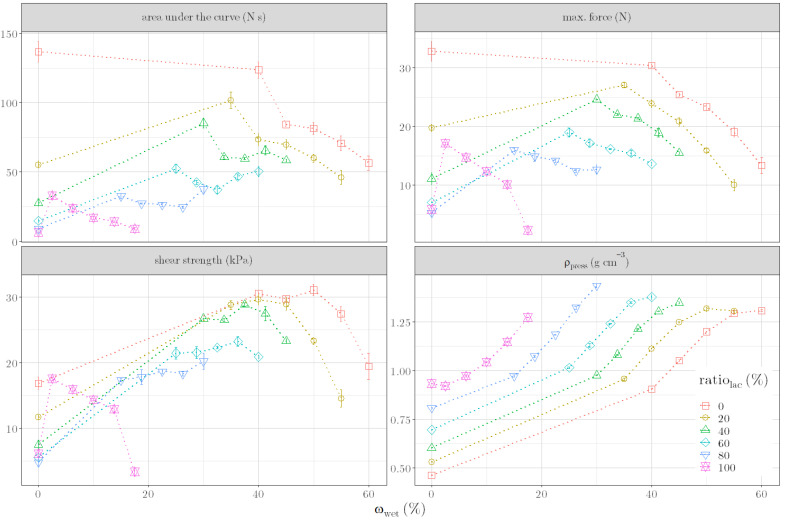
Area under the curve, max. force, shear strength and compression density (ρ_press_) versus ω_wet_ for formulations with 0%, 20%, 40%, 60%, 80% and 100% lactose ratio in the dry formulation (ratio_lac_).

Since we sought an alternative parameter for the quality of the granules related to their extrudability and spheronizability, it was expected that the parameters resulting from the shear of the granules would be suitable. However, experiments based on different formulations (Table A1) demonstrated that the area under the curve, max. force and shear strength are not suitable (Figure 2). Only ρ_press_ showed a clear dependence on both the formulation and moisture content.

In detail, the lactose ratio in the dry formulation (ratio_lac_) positively correlates with ρ_press_. This observation is in good agreement to the tapped density of two raw materials. For Lac 200, the tapped density is 0.82 g cm^−3^ [21] and the measured ρ_press_ is 0.93 g cm^−3^ (ratio_lac_ 100%). For MCC 101, the tapped density is 0.42 g cm^−3^ [22] and the measured ρ_press_ is 0.46 g cm^−3^ (ratio_lac_ 0%). It could be assumed that the higher the tapped density of the raw material, the higher ρ_press_ is. However, the tapped density is not exactly the same as ρ_press_. In both density measurements, particles are rearranged by externally applied forces (tapping or pressing). In the case of ρ_press_, the pressure can also lead to structural changes in particles due to breakage. Fractures due to applied pressure occur in brittle materials, such as lactose and mannitol. MCC, on the other hand, exhibits plastic deformation behavior and is not subject to fracture effects [23,24]. Thus, the differences between the tapped density and the measured ρ_press_ of 11% for Lac 200 and 9% for MCC 101 exhibit the higher compressibility of Lac 200 to MCC 101, which is caused by breakage during compression whereby small fragments fill the inter-particulate pore’s space.

**Figure 3:**
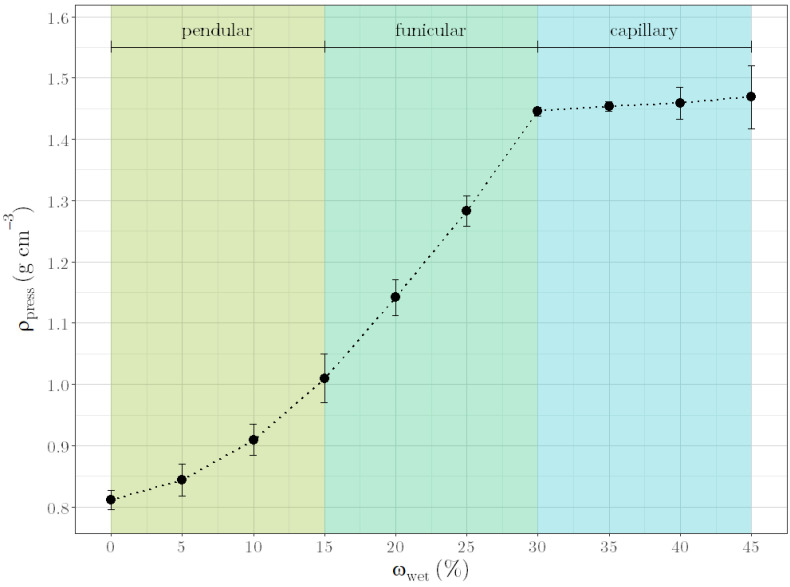
ρ_press_ versus ω_wet_ for a formulation with Lac 200 and MCC 101 in a ratio of 80:20. Granulation states are color annotated.

In detail, the dependence of ρ_press_ on ω_wet_ is shown in Figure 3. At a constant ratio_lac_, ρ_press_ increases with increasing ω_wet_, whereby a sigmoidal dependence can be recognized. After compressing the dry powder mixture, air-filled cavities are still present. These are filled with liquid when compressing moist granules, resulting in a higher ρ_press_. If all cavities are completely filled with liquid, ρ_press_ cannot increase further but remains constant. Related to the states of the granules described in the introduction, the graph in Figure 3 represents the pendular state in the first exponential section up to 15% ω_wet_. Here, intra-particulate pore spaces are initially filled with water, which only leads to minor changes in ρ_press_. At the transition from filling the intra-particulate pore spaces to filling inter-particulate pore spaces with liquid, the graph changes from the exponential section to the linear section. This section from 15% to 30% ω_wet_ represents the funicular state. The capillary state, where liquid saturation occurs, is described by the last section of the graph from 30% to 45% ω_wet_. This sigmoidal progression of ρ_press_ over ω_wet_ is similar to the change in power consumption with increasing moisture contents during granulation, as described by Leuenberger [7].

### 3.2. Prediction for a Standard Placebo Formulation with Different Ratios of Lac 200 and MCC 101 and Various Moisture Contents

Once ρ_press_ was found to show a correspondence with formulation compositions as well as with moisture content, the suitability of this alternative quality parameter should be verified by predicting the liquid requirement for further formulation compositions.

Based on the data from Figure 2, we used a multivariate regression model (Equation (3)) to estimate a relationship between ratio_lac_, ω_wet_ and ρ_press_ (*p* < 0.05).

(3) $$\begin{array}{cc} \rho_{\mathrm{press}} & =0.465+0.002785\;{\mathrm{ratio}}_{\mathrm{lac}}-0.06206\;\omega_{\mathrm{wet}}\\ \; & \;+0.0007451\;{\mathrm{ratio}}_{\mathrm{lac}}\;\cdot \;\omega_{\mathrm{wet}}+0.00001762\;{{\mathrm{ratio}}_{\mathrm{lac}}}^{2}\\ \; & \;+0.00294\;{\omega_{\mathrm{wet}}}^{2}-0.000001148\;{{\mathrm{ratio}}_{\mathrm{lac}}}^{2}\;\cdot \;\omega_{\mathrm{wet}}\\ \; & \;-0.00001393\;{\mathrm{ratio}}_{\mathrm{lac}}\;\cdot \;{\omega_{\mathrm{wet}}}^{2}-0.00002793\;{\omega_{\mathrm{wet}}}^{3} \end{array}$$

This model provided a coefficient of determination, *R*^2^, of 0.995 and a standard deviation of about 0.0178 g cm^−3^. The model is accurate, especially in the range of 0.95 g cm^−3^ < ρ_press_< 1.35 g cm^−3^, corresponding to realistic values.

For a typical placebo formulation in industry (80:20 Lac:MCC ratio, 25% ω_wet_), which shows good extrudability and spheronizability, a ρ_press_ of 1.26 gcm^−3^ was determined. Using Equation (3), we predicted the required *ω*_wet_ for three different so-far untested formulations to obtain a *ρ*_press_ of 1.26 g cm^−3^. Solving Equation (3) for *ω*_wet_ yields three solutions, two of which are discarded because their values are outside the valid range of *ω*_wet_. For Lac:MCC ratios of 30:70, 55:45 and 72:28, the valid solutions for *ω*_wet_ are 43.2%, 34.3% and 28.0%, respectively. The results of the experiments under predicted conditions are shown in Table 3.

The ρ_press_ of the three formulations resulting from the predicted water contents lie within the confidence interval of the prediction (Table 3). This indicates that the regression model is valid and that ρ_press_ is suitable for predicting liquid requirements for binary formulations of Lac 200 and MCC 101.

### 3.3. Proof of Concept for Standard Placebo Formulations

To show the applicability of *ρ*_press_ as an alternative measure of spheronizability, the processability of different formulations was assessed. For this purpose, the *ω*_wet_, required to obtain a *ρ*_press_ of 1.26 g cm^−3^, was predicted (Equation (3)) for three further formulations. We also predicted the required *ω*_wet_ for an over-wetted formulation at the upper limit of the valid range of Equation (3). Formulations and predicted *ω*_wet_ for the desired *ρ*_press_ are shown in Table 4.

**Figure 4:**
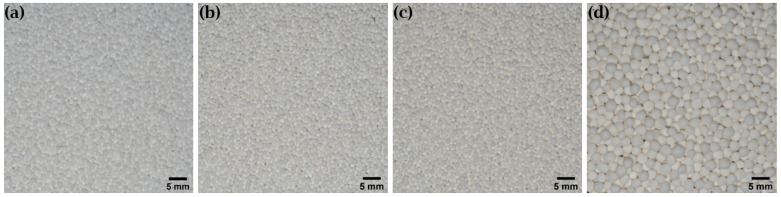
Pellets of the multi-step manufacturing process under predicted conditions. (**a**) Lac:MCC ratio 80:20, ω_wet_ 25.0%. (**b**) Lac:MCC ratio 70:30, ω_wet_ 28.7%. (**c**) Lac:MCC ratio 60:40, ω_wet_ 32.4%. (**d**) Lac:MCC ratio 60:40, ω_wet_ 35.9%.

**Figure 5:**
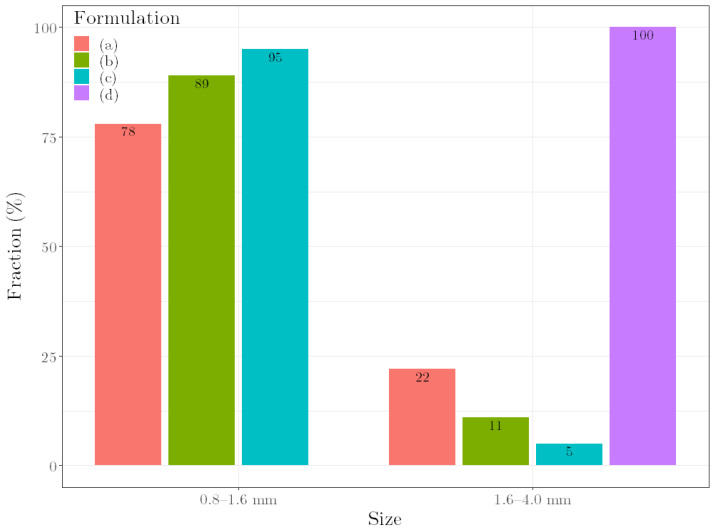
Particle size of the pellets of the different formulations. (**a**) Lac:MCC ratio 80:20, ω_wet_ 25.0%. (**b**) Lac:MCC ratio 70:30, ω_wet_ 28.7%. (**c**) Lac:MCC ratio 60:40, ω_wet_ 32.4%. (**d**) Lac:MCC ratio 60:40, ω_wet_ 35.9%.

The resulting pellets of the multi-step manufacturing process under predicted conditions are shown in Figure 4. For the formulations with the desired *ρ*_press_ of 1.26 g cm^−3^, the pellets are spherical and show a comparable average particle size (Figure 5). The slightly smaller size of the pellets with a higher MCC content is due to the behavior of the MCC, which tends to intrinsically round out [25]. In contrast, the pellets of the over-wetted formulation are spherical but show a much larger average particle size. This is due to coalescence, as over-wetted materials tend to uncontrolled agglomeration during spheronization, resulting in large pellets [3]. These results underscore that *ρ*_press_ is suitable for assessing the quality of pellets.

### 3.4. Applicability of ρ_press_ for Other Materials

**Figure 6:**
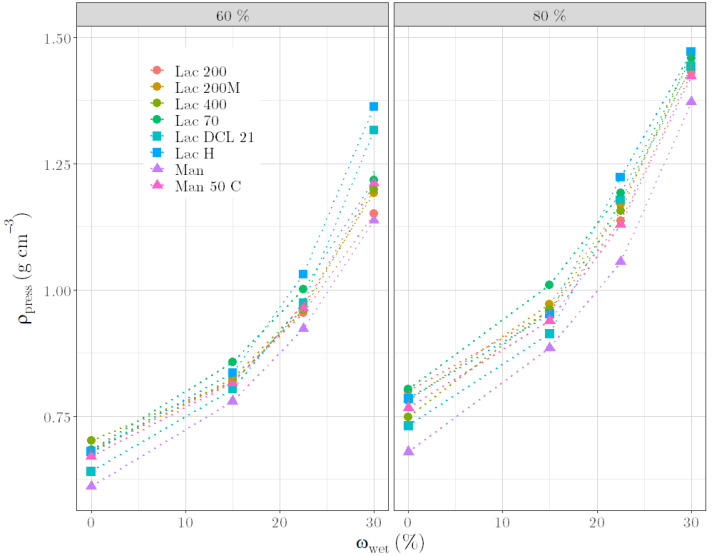
ρ_press_ versus ω_wet_ for formulations with different raw material A according to 60% (**left**) and 80% (**right**) content.

**Figure 7:**
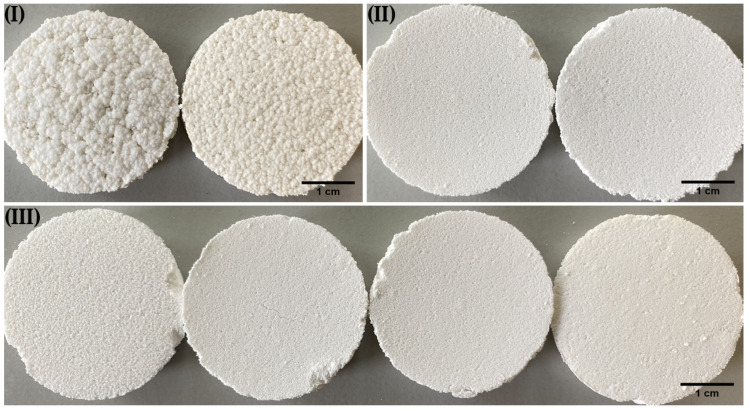
Surfaces of the briquettes with 60% ratio_alac_ and 30% *ω*_wet_. Left to right: (**I**) Lac H and Lac DCL 21. (**II**) Man and Man 50 C. (**III**) Lac 70, Lac 200, Lac 200M and Lac 400.

Changing raw material batches or brands during manufacturing can be critical, as small differences in the materials can already have a major impact on the quality of products. After demonstrating that *ρ*_press_ is a suitable quality parameter for formulations as a function of ratio_lac_ and *ω*_wet_, the applicability of the parameter to other placebo formulations should be investigated. It is noticeable that lactose monohydrate, lactose anhydrate and mannitol (raw materials A, Table 1) behave comparably with respect to the determined *ρ*_press_ at the same proportion in the formulation and the same *ω*_wet_ (Figure 6). This is in good agreement with the findings of Roberts and Rowe [26]. They stated that pure and water-free lactose, mannitol and also sucrose are materials with a medium yield pressure that are strain-rate dependent. This behaviour is indicative for moderately hard materials that plastically deform under loading. Only the *ρ*_press_ of formulations with 60% material A at 30% *ω*_wet_ show a differentiation between the material types. In detail, the anhydrous lactoses Lac H and Lac DCL 21 show the largest *ρ*_press_, and mannitol grades and lactose monohydrate grades have comparable *ρ*_press_. The similar compactibility behavior of formulations based on mannitol and lactose monohydrate is also visualized by the resulting surface of briquettes (Figure 7). However, as there is no significant difference between the *ρ*_press_ of dry formulations with Lac 400 and Lac 70 (both for 60% and for 80% ratio_lac_), the former assumption that *ρ*_press_ increases with an increasing tapped density of the raw materials must be put into perspective.

**Figure 8:**
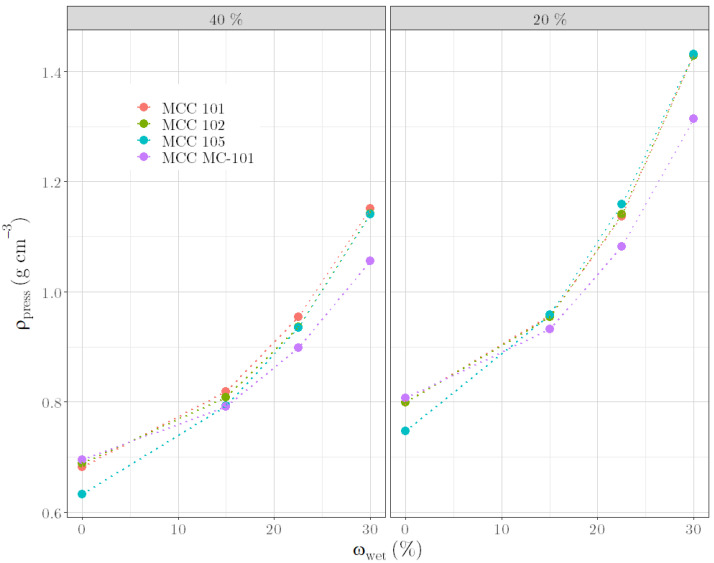
ρ_press_ versus ω_wet_ for formulations with different raw materials B according to 40% (**left**) and 20% (**right**) content.

Comparing different materials B (Figure 8), it is noticeable that the *ρ*_press_ of dry formulations differ insofar as the *ρ*_press_ for formulations with MCC 105 are distinctly lower than that of other formulations. This is caused by the very small particle size (the average particle size is 15 μm [27]) and the associated electrostatic effects, which decrease with water addition. However, this influence is minimized when water is added and the *ρ*_press_ converge to the same value with increasing water content. For *ω*_wet_ > 15%, the three VIVAPUR^®^ products (MCC 101, MCC 102, and MCC 105) behave comparably with respect to the determined *ρ*_press_ at the same proportion in the formulation. At contents of 20% and 40% and a *ω*_wet_ ≥ 22.5%, MCC MC-101 has a lower *ρ*_press_ than the other celluloses tested. This difference could result from the different manufacturer. MCC MC-101 is spray-dried [28], whereas the other celluloses are air-stream-manufactured [22]. These oberservations agree with Kleinebudde [3], who summarized that the particle size of MCC has only a minor effect on the liquid requirement, whereas different manufacturers and material sources have a much greater influence. In addition, other studies already described that celluloses of the same grade exhibit differences in their physicochemical properties [22,29].

### 3.5. Perspective for API Containing Formulations

**Figure 9:**
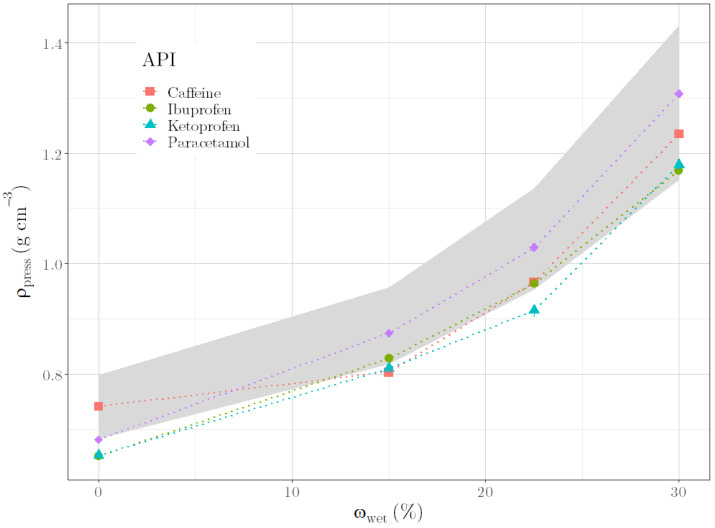
ρ_press_ versus ω_wet_ for ternary formulations with 40% API, 40% Lac 200 and 20% MCC 101. Highlighted in gray: range between the measured values of the binary formulation with Lac 200 and MCC 101 in a ratio of 60:40 (lower limit) and 80:20 (upper limit).

Pharmaceutical manufacturer typically produce API-containing formulations rather than placebo formulations. We thus performed first experiments to assess whether *ρ*_press_ could also be applied for API formulations. Again, we found that *ρ*_press_ increases with increasing *ω*_wet_, independent of the evaluated API formulations (Figure 9). For the purpose of alignment, the area highlighted in gray shows the range between the measured values of the placebo formulation with Lac 200 and MCC 101 in a ratio of 60:40 (lower limit) and 80:20 (upper limit). The data of the ternary formulations lie in the highlighted area or slightly below. This shows that even with the use of APIs, the values of *ρ*_press_ have a comparable order of magnitude. For a more accurate assessment, the API content in the formulation has to be increased in further trials and additional APIs have to be tested. This first insight nevertheless indicates the possibility of transferring the diagnostic ability of the *ρ*_press_ measurement to ternary formulations with APIs.

### 3.6. Proof of Concept for Other Materials

As an example, the pellet’s production in lab-scale was carried out for formulations with Lac 400 (representative for lactose monohydrate), Lac DCL 21 (representative for lactose anhydrate) and Man 50 C (representative for mannitol) with MCC 101 at a ratio of 60:40. Representative for API, a formulation with Paracetamol at a ratio of 40:40:20 with Lac 200 and MCC 101 was used. We used the datapoints of *ρ*_press_ at 22.5% and 30% water content (Figure 6 and Figure 9) to calculate *ω*_wet_ required to achieve a *ρ*_press_ of 1.26 g cm^−3^. Formulations and the estimated values for *ω*_wet_ for the desired *ρ*_press_ are shown in Table 5.

**Figure 10:**
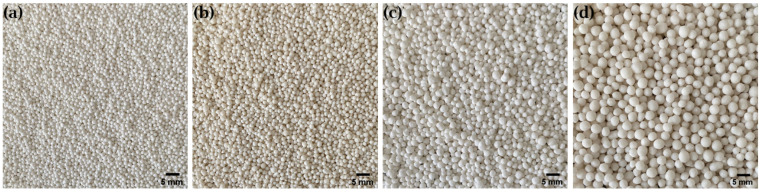
Pellets of the multi-step manufacturing process. (**a**) Lac 400:MCC 101 ratio 60:40, ω_wet_ 31.9%. (**b**) Lac DCL 21:MCC 101 ratio 60:40, ω_wet_ 28.8%. (**c**) Man 50 C:MCC 101 ratio 60:40, ω_wet_ 31.5%. (**d**) Paracetamol:Lac 200:MCC 101 ratio 40:40:20, ω_wet_ 28.7%.

The resulting pellets of the multi-step manufacturing process, using the parameter values calculated above, are shown in Figure 10. The formulation with lactose monohydrate shows the smallest pellet size, followed by lactose anhydrate and mannitol. An effect of the particle size of the applied materials (see Table 1 and information in the product data sheets) on the quality of the pellets could not be observed. However, it appears that the increase in the strain rate’s sensitivity index [26] directly correlates with the increase in pellet size. The formulation with paracetamol shows the largest pellets. This finding is not in agreement with the strain rate sensitivity index presented by Roberts and Rowe [26], which might be due to the fact that they analyzed spray-dried Paracetamol with 4% hydrolised gelatin. However, Paracetamol in contrast to lactose, and mannitol is regarded as highly elastic [30]. As it was noticed during extrusion that all four formulations extrude very well and smoothly, straight strands were produced, and a smaller diameter of the extrusion die could have led to an acceptable pellet size for Paracetamol-loaded pellets. Thus, it can be assumed that, for different formulations, a *ρ*_press_ of 1.26 g cm^−3^ is a suitable indicator for the extrudability of the granules. In summary, a *ρ*_press_ of 1.26 g cm^−3^ can be regarded as a type of anchor for good extrudability, from which the fine adjustment of the moisture content and/or the equippment can be made depending on the desired pellet size.

## 4. Conclusions

To evaluate the quality of the granules during granulation, the *ρ*_press_ of the granule mass was identified as an alternative parameter. This parameter showed a relation with formulation compositions as well as with moisture content. A regression model, based on this relation, was successfully used to predict liquid requirements for unknown formulation compositions. In addition, uniform pellets were produced by carrying out the multi-step manufacturing process of granulation, extrusion and spheronization with the predicted liquid requirements for different formulations. Since these results could verify the suitability of *ρ*_press_ as an alternative quality parameter, the applicability of the parameter to other placebo formulations was demonstrated. Moreover, first experiments with API formulations suggested the applicability to industrially relevant formulations. In conclusion, the results indicate that once a *ρ*_press_ is defined for an optimal formulation, changes in material batches and even material types could be compensated with the right amount of water. However, one remaining question is which material properties, in addition to the tapped density and the brittleness, influence *ρ*_press_ or correlate with it. If defined material properties could be determined, *ρ*_press_ would not have to be defined for an optimal formulation but could be determined generally for corresponding material properties. One remaining disadvantage of the presented method is that the optimal *ρ*_press_ for ideal further processing depends on the process parameters of the further processing itself (such as the extruder die). Therefore, no generally valid statement can be made about an optimal *ρ*_press_; this must be individually adapted to the multi-step manufacturing process’ parameters used.

## Figures and Tables

**Table 1:** Raw materials used in this study.

Material		Type	Abbreviation	Company
A	Lactose anhydrate	DuraLac^®^ H	Lac H	Meggle, Wasserburg, Germany
		Pharmatose^®^ DCL 21	Lac DCL 21	DMV International, Veghel, The Netherlands
	Lactose monohydrate	GranuLac^®^ 70	Lac 70	Meggle, Wasserburg, Germany
		GranuLac^®^ 200	Lac 200	Meggle, Wasserburg, Germany
		Pharmatose^®^ 200M	Lac 200M	DFE Pharma, Goch, Germany
		SorboLac^®^ 400	Lac 400	Meggle, Wasserburg, Germany
	Mannitol	D(-)-Mannit	Man	Merck, Darmstadt, Germany
		PEARLITOL^®^ 50 C	Man 50 C	Roquette Frères, Lestrem, France
B	Microcrystalline cellulose	Microcel^®^ MC-101	MCC MC-101	Roquette Frères, Lestrem, France
		VIVAPUR^®^ 101	MCC 101	JRS Pharma, Rosenberg, Germany
		VIVAPUR^®^ 102	MCC 102	JRS Pharma, Rosenberg, Germany
		VIVAPUR^®^ 105	MCC 105	JRS Pharma, Rosenberg, Germany
API	Active pharm. ingredient	Caffeine (pure)		Merck, Darmstadt, Germany
		(2.0 μm ± 0.4 μm; *n* = 17) *		
		Ibuprofen 25		BASF, Ludwigshafen, Germany
		(52.4 μm ± 25.9 μm; *n* = 17)		
		Ketoprofen		Hubei Xunda Pharmaceutical, Wuxue, China
		(2.8 μm ± 0.6 μm; *n* = 17)		
		Paracetamol		Merck, Darmstadt, Germany
		(19.5 μm ± 15.6 μm; *n* = 48)		

* Mean particle size with standard deviation and sample size (*n*). Particle size was measured by suspending the sample in paraffin and measuring it using a microscope (Olympus BX41).

**Table 2:** Experimental procedure. Abbreviations and labeling can be found in Table 1.

Combination of Raw Materials	Raw Material Ratio	ω_wet_ *
Lac 200:MC 101 ^1^	0:100–100:0	see Table A1
Lac 200:MC 101 ^2^	80:20	0–45%
A:MCC 101	80:20 and 60:40	0%, 15%, 22.5%, 30%
Lac 200:B	80:20 and 60:40	0%, 15%, 22.5%, 30%
API:Lac 200:MCC 101	40:40:20	0%, 15%, 22.5%, 30%

* *ω*_wet_ is the water content on wet basis. ^1^ Formulation with 100% Lac 200: use of 180 g dry mass instead of 150 g. ^2^ Three batches; each batch *n* = 1.

**Table 3:** Obtained ρ_press_ as means with corresponding standard deviations (SD) and confidence intervals of the experiments under predicted conditions.

Lac:MCC Ratio	ρ_press_ (g cm^−3^)		
	Mean (n=3)	SD	95%-Confidence Interval
30:70	1.262	±0.00393	[1.252; 1.268]
55:45	1.264	±0.00318	[1.254; 1.266]
72:28	1.253	±0.00130	[1.253; 1.267]

**Table 4:** Predicted *ω*_wet_ to obtain desired *ρ*_press_ for different formulations.

Formulation	Lac:MCC Ratio	Desired *ρ*_press_ (g cm^−3^)	Predicted *ω*_wet_ (%)
(a)	80:20	1.26	25.0
(b)	70:30	1.26	28.7
(c)	60:40	1.26	32.4
(d)	60:40	1.34	35.9

**Table 5:** Estimated ω_wet_ to obtain a ρ_press_ of 1.26 g cm^−3^ for different formulations.

Formulation	Estimated ω_wet_ (%)
(a) 60% Lac 400, 40% MCC 101	31.9
(b) 60% Lac DCL 21, 40% MCC 101	28.8
(c) 60% Man 50 C, 40% MCC 101	31.5
(d) 40% Paracetamol, 40% Lac 200, 20% MCC 101	28.7

## Data Availability

Not applicable.

## Abbreviations

The following abbreviations are used in this manuscript:

API	active pharmaceutical ingredient
ρ _press_	compression density
ratio_lac_	lactose ratio in the dry formulation
ω _wet_	water content on wet basis

## Appendix A

**Table A1:** Raw data corresponding to Table 2 and Figure 2. The ratios of the raw materials Lac 200 and MCC 101 refer to the total dry mass, whereas the water content ω_wet_ refers to the total wet mass.

Lac 200 (%)	MCC 101 (%)	*ω*_wet_ (%)
0	100	0
		40
		45
		50
		55
		60
20	80	0
		35
		40
		45
		50
		55
40	60	0
		30
		33.75
		37.5
		41.25
		45
60	40	0
		25
		28.75
		32.5
		36.25
		40
80	20	0
		15
		18.75
		22.5
		26.25
		30
100	0	0
		2.5
		6.25
		10
		13.75
		17.5

**Table A2:** Determined residual moisture contents of the dried pellets corresponding to Table 4, Figure 4 and Table 5, Figure 10.

Formulation	ω_wet_ (%)	Residual Moisture (%)
80% Lac 200, 20% MCC 101	25.0	0.39
70% Lac 200, 30% MCC 101	28.7	0.79
60% Lac 200, 40% MCC 101	32.4	0.40
60% Lac 200, 40% MCC 101	35.9	0.10
60% Lac 400, 40% MCC 101	31.9	0.31
60% Lac DCL 21, 40% MCC 101	28.8	0.16
60% Man 50 C, 40% MCC 101	31.5	0.22
40% Paracetamol, 40% Lac 200, 20% MCC 101	28.7	0.10
